# Empty follicle syndrome—Still an enigma

**DOI:** 10.4103/0974-1208.44118

**Published:** 2008

**Authors:** Deepika Krishna, Lavanya Rajashekar, Madhuri Patil

**Affiliations:** O.B.G-Infertility, Dr. Patil's Fertility and Endoscopy Clinic, No1, Uma Admiralty, 1^st^ Floor, Banerghatta Road, Bangalore-560 029, Karnataka, India

**Keywords:** Beta HCG, controlled ovarian hyperstimulation (COH), empty follicle syndrome (EFS), oocyte retrieval (OR)

## Abstract

Empty follicle syndrome (EFS), although rare with an incidence of 0.2–7%, is a frustrating condition where no oocytes are retrieved in in vitro fertilization (IVF), even though ultrasound and estradiol measurements show the presence of many potential follicles. It is a complex phenomenon that cannot be explained by low bioavailability of human chorionic gonadotrophin alone; neither can it be reliably diagnosed by the measurement of serum beta-human chorionic gonadotrophin (bhCG) on the day of oocyte retrieval (OR), except possibly when the bhCG concentration is very low. Here we report a case who underwent intracytoplasmic sperm injection (ICSI) for her partner's severe oligoasthenozoospermia. Controlled ovarian hyperstimulation (COH) was done in her first cycle of ICSI, using a gonadotrophin-releasing hormone (GnRH) agonist long protocol with follicle-stimulating hormone (FSH) and human menopausal gonadotrophin (HMG). However, as we were unable to retrieve any oocytes, her COH protocol was changed in the subsequent cycle with a successful outcome.

## INTRODUCTION

A failure to collect oocytes after an apparently normal controlled ovarian hyperstimulation (COH) cycle for *in vitro* fertilization (IVF) has caught the attention of many clinicians. This feature could be traumatic, for both the couple and the clinical staff involved.[1]

Empty follicle syndrome (EFS) has been defined as a condition in which no oocytes are retrieved from mature ovarian follicles with apparently normal follicular development and estradiol levels, after COH for an assisted reproductive technology (ART) cycle, despite repeated aspiration and flushing.

It is evident from a review of literature that two types of EFS exist: genuine (GEFS) and false (FEFS). About 67% of all cases are due to human error, suggesting that GEFS is an even rarer event than previously presumed.[2] Here we report a case of GEFS, which was managed successfully in the subsequent cycle by varying the stimulation protocol.

## CASE REPORT

Our patient was Mrs. SR, a 23 year-old woman with primary infertility for three years, who underwent intracytoplasmic sperm injection (ICSI) at our center for her partner's severe oligoasthenozoospermia. She had normal menstrual cycles with no significant past history. Both her parents were diabetic and her twin sister had undergone two IVFs for unexplained infertility with a poor response (two oocytes in one cycle and three in the second) and no conception. Her routine investigations, glucose tolerances test (GTT) and hysterosalphingography were normal. Her day 2 hormonal profile: levels of follicle-stimulating hormone (FSH), luteinizing hormone (LH), estradiol (E2) and progesterone (P4) were all within normal limits. Hysteroscopy done on day 6 revealed thick hyperplastic endometrium with multiple polyps which were removed and histopathological examination showed a progesterone response of the endometrium.

Ovarian stimulation was accomplished using a GnRH agonist long protocol. An oral contraceptive was given from days 2 to 22 of the cycle and a GnRH agonist (Lupride, Sun Pharmaceutical Ltd) at a dose of 250 micrograms, twice daily from day 21, after a transvaginal ultrasound was done that confirmed no cysts. This protocol was continued until day 2 of the cycle when the levels of E2 and P4 were 19.56 pg/mL and 0.77 ng/mL respectively. Thereafter, the GnRH agonist was given once a day along with gonadotrophins: FSH (Bravelle, Ferring Pharmaceuticals) 150 IU and HMG (Nugon, Solvay pharma) 75 IU. As the levels of E2 on days 4 and 7 of the COH were 46 and 104 pg/mL respectively, the dose of hMG was increased to 150 IU from day 8 of the COH. On day 10 of the COH, the E2 level was 1774 pg/mL. On day 13 of the COH, recombinant hCG (Ovitrelle, Serono) at a dose of 250 micrograms was given subcutaneously. On the day of hCG administration, the E2 level was 2457 pg/mL and the endometrial thickness was 14.9 mm with eight dominant follicles > 17 mm in diameter. Thirty-six hours later, all the follicles were transvaginally aspirated under sonographic guidance. As no oocytes were obtained in the first two tubes, the remaining follicles were flushed after changing the aspiration needle to one with a double lumen. In spite of flushing, there was only follicular fluid with granulosa cells and as we did not obtain any oocytes, we measured the serum beta hCG and progesterone concentrations which were 182 IU/L, 7.2 ng/mL, respectively. These findings were perplexing and the authors then considered whether measurement of the LH levels along with E2 levels would have helped on the day of hCG administration in determining a premature LH surge due to problems related to hCG or GnRH agonist.

**Figures 1:**
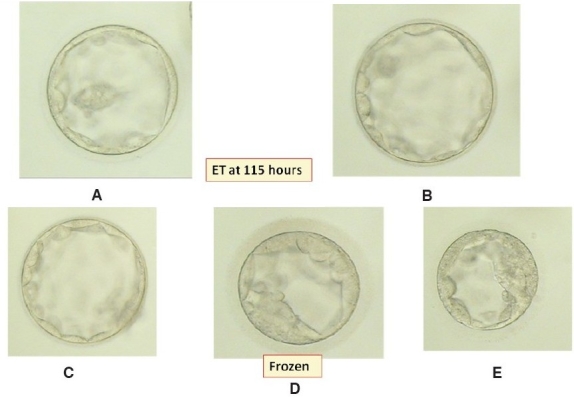
ET at 115 hours (A) Grade 4 BA (B) Grade 3 BA (C) Grade 4 BA (D) Grade 2 (E) Grade 2

In the subsequent cycle, an antagonist protocol was planned with gonadotrophins. The patient's day 2 hormonal profiles were normal. COH was started with FSH (Bravelle, Ferring Pharmaceuticals) at a dose of 150 IU and HMG (Nugon, Solvay Pharma) at a dose of 75 IU from day 2 onwards. The GnRH antagonist orgalutron (Ganirelix organon Ltd) was started at a dose of 0.25 mg when the dominant follicle size was 14 mm. E2 levels measured on days 5 and 8 were 221 and 900 pg/mL respectively. On the day of hCG (day 11), the endometrial thickness was 16.5 mm and this time, we measured LH levels along with those of E2: 1.2 mIU/mL and 2397 pg/mL respectively. Recombinant hCG (Ovitrelle, Serono) was given subcutaneously at a dose of 500 micrograms when there were ten follicles > 18 mm in size. OR was done 36 hours later wherein13 oocytes were obtained. All MII and ICSI were done with 100% fertilization rate. Five blastocysts were formed: two of Grades 4BA and 3BA [Figures 1A and B] were transferred and the remaining three were frozen [Figures 1C–E].

Luteal support was given with injectable progesterone (Gestone, Ferring Pharmaceuticals) at a dose of 100 mg IM daily. Fifteen days after embryo transfer (ET), the beta hCG level was 435 mIU/mL but 21 and 28 days after ET, these levels were found to have increased to 3855 and 10,070 mIU/mL respectively. A live intrauterine pregnancy was confirmed by USG. As the patient had a family history of diabetes, a glucose tolerance test (GTT) was done at eight weeks and as the results were outside normal limits, insulin was started. Apart from diabetes, the patient did not have any other complications and at 36 weeks, she delivered by cesarean section, a female baby weighing 2500 g.

## DISCUSSION

EFS first reported by Coulam *et al*.[3] in 1986, may not strictly be a syndrome, but a sporadic unpredictable event.[4] It cannot be predicted by the pattern of ovarian response to stimulation, either sonographically or hormonally. Consequently, the diagnosis of EFS is retrospective.

GEFS is defined as a failure to retrieve oocytes from mature follicles after apparently normal folliculogenesis and steroidogenesis with optimal beta hCG levels on the day of OR. Such patients are unlikely to respond to a rescue protocol. FEFS is defined as a failure to retrieve oocytes in the presence of low bhCG due to an error in the administration or bioavailability of hCG. Such patients are more likely to respond to a rescue protocol. Moreover, the condition of FEFS should not recur, provided caution is exercised in subsequent cycles.[2]

Since the original description by Coulam *et al*., most clinicians have experienced EFS in patients having various infertility factors. It was initially suggested that EFS might stem from the same cause that is responsible for the patient's infertility.[3] Various hypotheses have been put forth ranging from human error[5–7] to pharmacological problems.[7–9] Possible etiologies for EFS include: (1) Dysfunctional folliculogenesis, in which early oocyte atresia occurs with apparently normal hormonal response,[4] (2) Biological abnormality in the supply of mature oocytes that can be retrieved, despite normal bioavailability of hCG,[7] (3) Genetic factors in some cases,[10] (4) Drug-related causes due to an abnormality in the in vivo biological activity of some batches of commercially available hCG [6] or GnRH agonist; inappropriate timing of hCG,[8] or rapid clearance of hCG by the liver,[6] and (5) Advanced ovarian ageing through altered folliculogenesis.[10]

EFS does not predict a reduced fertility potential in future cycles, although it may recur due to a biological abnormality in the availability of mature oocytes that can be retrieved, especially in advanced aged patients. If EFS has occurred once, the risk of recurrence is 20%,[10] the risk being higher with advancing age. The risk of recurrence in patients between 35 and 39 years of age has been reported to be 24 whereas it is 57% for those > 40 years of age.[10] In such patients, oocyte donation may offer a chance of achieving a pregnancy.

The surge of the surrogate leutinizing hormone (LH) due to administration of hCG plays a crucial role in intrafollicular events such as softening of the connective tissue elements, facilitating the detachment of the oocyte-cumulus complex from the follicle wall, resumption of meiosis with extrusion of first polar body and subsequent ovulation. The threshold level of the LH (hCG) surge is not known for certain, but various authors have reported the lowest concentration when oocytes were successfully retrieved following COH. Zegers-Hochschild reported a value of 110 mIU/mL in 1995.

Ndukwe *et al*. reported a value of 106 mIU/mL in 1996, but in 1997, they reported that serum beta hCG concentrations of < 10 mIU/mL prevent preovulatory changes within the follicle; sensitivity, specificity and predictive values were all 100%.[8] Stevenson *et al*. published a cut-off level of < 40 mIU/mL for beta hCG 36 hours after the hCG injection.[11] Thus, it is easy to understand those cases of EFS that occur due to the absence of any hCG injection.

The importance of a temporal relationship between the administration of hCG and OR has been emphasized in literature.[10] If OR is attempted too early, a repeat aspiration can be successfully carried out at the more appropriate interval of 36 hours after the hCG injection.[10]

Apart from these human errors, it is now well accepted that decreased hCG availability, whatever its origin, seems to be the fundamental cause of poor OR in many cases.[6] It has been demonstrated that EFS can also be due to the rapid clearance of the hCG that was injected due to some defect arising during the production, packaging or storage of a particular batch of the drug. Thus, the rapid clearance of the drug by the liver would prevent the exposure of relevant follicular processes to the action of HCG. On this basis, a rescue protocol can be used to salvage the cycle when the beta hCG concentrations are < 100 mIU/mL (Zegers-Hochschild); < 10 mIU/mL (Ndukwe *et al*. ) or < 40 mIU/mL (Stevenson *et al*.) 36 hours after the hCG injection when no oocytes are obtained from the follicles in one ovary. Administration of a second ovulatory injection of hCG from a different batch and rescheduling OR 24–36 h later would yield mature oocytes from the intact ovary.[6,8,11]

EFS does not represent a permanent pathophysiological condition and most cases occur only sporadically. Cycles with EFS have been reported to be preceded or followed by cycles with successful oocyte retrieval,[6,9] as is evident in our patient.

In our patient who had normal folliculogenesis and steroidogenesis with optimal levels of beta hCG on the day of OR, the probable cause of EFS could have been insufficient exposure of the oocytes to the biologically active hCG or a premature LH surge due to reduced bioactivity of the GnRH agonist used for downregulation.[8] Either the dose of hCG injected had insufficient bioactivity or the ovaries showed an insufficient or delayed response to the administered dose.[9] A genetic predisposition cannot be ruled out as her twin sister also had a poor response to COH in two IVF cycles with no pregnancy. We did not find it necessary to inject hCG from a different batch on a second occasion as the beta hCG on the day of OR was optimal and was administered by qualified personnel and OR done 36 h later.

In the next cycle, an antagonist protocol was used as the majority of EFS cases reported to date are with agonist protocol; a higher dose of recombinant hCG from a different batch was used.

Strategies suggested to prevent the occurrence of EFS in a subsequent ART cycle are: (1) Using recombinant hCG to trigger an endogenous LH surge, (2) Changing the batch of hCG, (3) Use of an antagonist protocol, (4) Using a GnRH agonist in an antagonist cycle to induce LH surge, (5) A rescue protocol by administrating a second dose of hCG and rescheduling OR 24–36 h later and (6) EFS related to timing of hCG; a rescheduled follicle puncture is feasible.

To conclude, ovarian follicles of patients with so-called EFS may not actually be devoid of viable oocytes. The problem seems to be that of inadequate preovulatory follicular changes arising from either poor bioavailability of LH or hCG or too short an interval between the onset of these changes and follicular aspiration. Premature lutenization due to a premature LH surge and high progesterone levels on the day of hCG injection, can also effect the oocyte recovery.

EFS does not predict a reduced fertility potential in future cycles. Nevertheless, whatever the cause of EFS, these patients should be counselled regarding its possibility of recurrence and future poor prognosis. Different IVF treatment methods in subsequent cycles could also modulate the response with successful oocyte recovery in such cases.
